# The Cerebro-Cerebellum as a Locus of Forward Model: A Review

**DOI:** 10.3389/fnsys.2020.00019

**Published:** 2020-04-09

**Authors:** Hirokazu Tanaka, Takahiro Ishikawa, Jongho Lee, Shinji Kakei

**Affiliations:** ^1^Japan Advanced Institute of Science and Technology, Nomi, Japan; ^2^Tokyo Metropolitan Institute of Medical Science, Tokyo, Japan; ^3^Komatsu University, Komatsu, Japan

**Keywords:** cerebral cortex, cerebellar circuitry, forward model, motor function, higher brain function, neural networks

## Abstract

This review surveys physiological, behavioral, and morphological evidence converging to the view of the cerebro-cerebellum as loci of internal forward models. The cerebro-cerebellum, the phylogenetically newest expansion in the cerebellum, receives convergent inputs from cortical, subcortical, and spinal sources, and is thought to perform the predictive computation for both motor control, motor learning, and cognitive functions. This predictive computation is known as an internal forward model. First, we elucidate the theoretical foundations of an internal forward model and its role in motor control and motor learning within the framework of the optimal feedback control model. Then, we discuss a neural mechanism that generates various patterns of outputs from the cerebro-cerebellum. Three lines of supporting evidence for the internal-forward-model hypothesis are presented in detail. First, we provide physiological evidence that the cerebellar outputs (activities of dentate nucleus cells) are predictive for the cerebellar inputs [activities of mossy fibers (MFs)]. Second, we provide behavioral evidence that a component of movement kinematics is predictive for target motion in control subjects but lags behind a target motion in patients with cerebellar ataxia. Third, we provide morphological evidence that the cerebellar cortex and the dentate nucleus receive separate MF projections, a prerequisite for optimal estimation. Finally, we speculate that the predictive computation in the cerebro-cerebellum could be deployed to not only motor control but also to non-motor, cognitive functions. This review concludes that the predictive computation of the internal forward model is the unifying algorithmic principle for understanding diverse functions played by the cerebro-cerebellum.

## Introduction

The cerebellum has developed in the sensory domain of the central nervous system (CNS) as evidenced by the fact that it has emerged in the alar plate (i.e., the sensory part (dorsal half) of the neural tube of the rhombencephalon of primitive jawless vertebrate such as myxinoids (hagfish) or petromyzonts (lampreys; Larsell, 1967; Sugahara et al., 2016). The cerebellum is hence ideally located to accommodate multimodal sensory inputs (Larsell, 1967) including both exteroceptive (lateral-line, vestibular, acoustic, visual) and interoceptive (somatosensory) inputs, thereby functioning as the hub of sensory integration. In addition to sensory inputs, the mammalian cerebellum receives inputs from cortical areas including sensory, motor, and association areas through the pontine nuclei (PN). In humans, the cerebellum is estimated to share no less than 80% of the CNS neurons in no more than 10% of the brain volume (Herculano-Houzel, 2009). To summarize, the cerebellum has been, throughout its long phylogenetic history, the unique hub in the CNS to accommodate and integrate both afferent and efferent inputs from almost the entire brain, a prerequisite for a neural substrate for adaptive and flexible behaviors.

The cerebellum is homogenous in its local neural circuity and heterogeneous in its input-output organization (Ito, 1984). The local neural circuitry of the cerebellar cortex is characterized by its superb homogeneity, sometimes expressed as being “crystal-like,” whereas the inputs to and the outputs from the cerebellum are heterogeneous from one region to another. Therefore, it is commonly postulated that the functional diversity of the cerebellum originates from heterogeneous input-output connectivity and is processed through a common algorithm that is implemented in the homogenous neural circuitry in the cerebellar cortex. In light of Marr’s three levels of analysis, the cerebellum may perform common computation on diversified representations of cerebellar inputs and outputs. Therefore, the cerebellum may be better understood if we ask “how the cerebellum computes” than “what the cerebellum computes.” In contrast to previous studies that have focused on neural representations of an internal forward model, we explored how the cerebellum transforms its inputs [mossy fibers (MFs)] to its outputs [dentate nucleus cells (DNCs); Tanaka et al., 2019]. Given the postulate that the cerebellum may process a common algorithm, the main goal of this review is to comprehend the physiology and pathology of different regions of the cerebellum on a common ground of the algorithm (Diedrichsen et al., 2019).

Among a range of proposals for the cerebellar algorithm including timing processing (Ivry et al., 2002; Ivry and Spencer, 2004) and temporal pattern generator (Fujita, 1982; Dean et al., 2010), one plausible candidate is the prediction of sensory outcomes as a consequence of motor action, referred to as the computation of an internal forward model (Jordan and Rumelhart, 1992; Wolpert and Miall, 1996; Bastian, 2006; Ishikawa et al., 2016). The predictive computation of the internal forward model plays a key role in predicting outcomes of self-action, fast and stable motor control, integrating the prediction with sensory feedback, and adaptation to a novel environment. This review article provides physiological, behavioral, and morphological evidence that converges to the cerebro-cerebellum as a neural substrate of the internal forward model. In particular, we introduce neural evidence that current outputs from the cerebellum [i.e., outputs from the dentate nucleus (DN)] can predict future inputs to the cerebellum (i.e., cortical outputs relayed by MFs originated from PN), a hallmark of an internal forward model. We will also discuss how the input-output organization of the cerebro-cerebellum may contribute to forward models for higher (i.e., non-motor) brain functions.

This article is organized as follows. Section “Predictive Computation of Internal Forward Model” begins by defining the computation of the internal forward model, discusses its multiple functions in a computation model of motor control and motor adaptation, and reviews experimental evidence for the cerebellum as a locus of an internal forward model. Whereas the topic of internal models has been reviewed previously in the existing literature, this section emphasizes background information for facilitating the following discussions. In particular, we emphasize multiple computational roles that an internal forward model can play. Section “Generation of Outputs From the Cerebro-Cerebellum” delves into a physiological mechanism to generate a wide range of output from DN by modulating the inhibitory inputs from Purkinje cells (PCs). Section “Functional Evidence for Cerebellar Forward Model” summarizes neural and behavioral evidence that the cerebellum performs predictive computation. Section “Anatomical Structure Supporting for Cerebellar Forward Models” surveys anatomical structures of the cerebro-cerebellum that potentially scaffold the forward-model computation. Section “Cognitive Functions and Cerebellar Forward Models” branches out to speculate a possible role of the cerebro-cerebellum in higher cognitive functions from the viewpoint of the internal forward model. Finally, Section “Remaining Issues About Cerebellar Internal Models” concludes the internal-model hypothesis of the cerebellum and enumerates unresolved issues toward the goal of understanding the cerebro-cerebellum.

## Predictive Computation of Internal Forward Model

### Internal Forward Model and its Computational Roles

One critical problem in biological motor control is that afferent sensory signals have inevitable temporal delays in reaching the central nervous system. In other words, the brain always observes “the past” of its own body and environments. Visual signals, for example, arrive at the primary visual cortex about 30 ms later and at the parietal cortex about 80 ms later than an onset of a visual stimulus (Schmolesky et al., 1998). Delays in sensory feedback originate from several factors, as quantified in locomotor reflex movements in terrestrial animals of various sizes (More et al., 2010; More and Donelan, 2018). Among factors contributing to the feedback delay such as a synaptic delay or an electro-mechanical delay, the dominant factor is the nerve conduction delay, ranging about 10 ms for a shrew to about 100 ms for an elephant. Larger animals experience a longer feedback delay yet move more slowly, whereas smaller animals experience a shorter feedback delay but move more quickly. In sum, sensory delays are comparable to typical time scales of rapid movements and hence not negligible both in small and large animals.

The delay in sensory feedback is problematic not only in sensing the body and the environments but also in controlling the body. It is well known in control engineering that feedback control based on a previous state causes oscillatory and unstable movements if the delay in feedback control is of the order of or larger than a time constant of a controlled plant (Wolpert and Miall, 1996). The delays in visual feedback are comparable to the movement time of rapid reaching movement of the upper limb (about a few hundred milliseconds) and saccadic eye movements (typically less than fifty milliseconds). Therefore, in biological motor control, feedback control based on delayed sensory signals would result in unstable movements. Nonetheless, animals can perform a fast movement without losing its stability. Biological motor control must be equipped with a mechanism to compensate for the sensory delay for a fast and stable movement.

**Figure 1:**
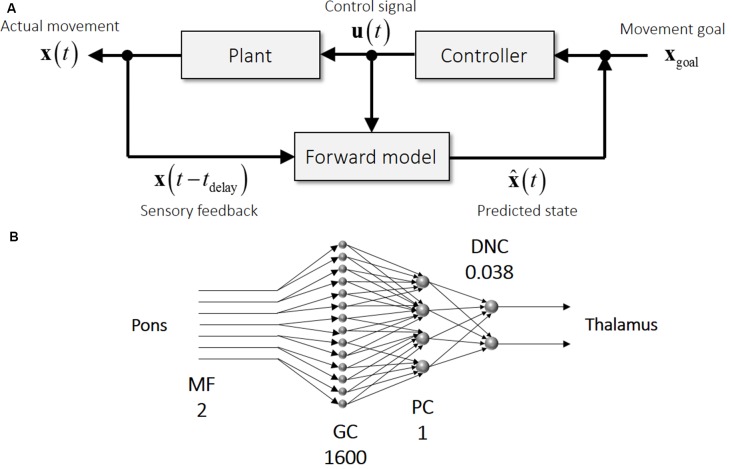
Schematics of motor control and the cerebellar circuit. **(A)** An internal forward model receives time-delayed sensory feedback (**x**_(*t* − *t* delay)_ and efference copy (**u** (t)) and predicts a current state ($\overset{⌢}{x}$ (t)) (bottom row). Difference between movement goal (**x**_goal_) and predicted state ($\overset{⌢}{x}$ (t)) drives a feedback controller to generate a control signal (**u** (t)), which in turn steers the controlled plant (top row). **(B)** Schematics of the neural circuit in the cerebellum from mossy fibers (MFs) to dentate nucleus cells (DNCs). Note that this feedforward circuitry resembles that of an internal forward model, indicating that the cerebellar input representing a time-delayed state is converted into the cerebellar output representing a current state. The numbers accompanied by the cell labels denote the relative numbers of cells in comparison with Purkinje cells (PCs), illustrating the expansion from MFs to granule cells (GCs) and the compression from MFs to DNCs. These numbers were calculated from corresponding numbers listed in Ito (1984).

One mechanism proposed to cope with the delay in sensory feedback is to compute a future state of the body based on a current estimate of the body and an efferent signal of motor control (Figure 1A). This predictive computation internally emulates or models an actual movement of the body by essentially solving an equation of motion of the body forward in time, thereby known as an internal forward model (Wolpert et al., 1998; McNamee and Wolpert, 2019). An internal forward model predicts the state of the body time by time that is then used by a feedback controller, thereby allowing fast and stable movements. The feedback control based on the prediction of the internal forward model is called internal feedback. There are lines of evidence supporting the hypothesis of predictive forward model and internal feedback from neuroimaging studies (Heinks-Maldonado et al., 2006; Bäss et al., 2008), non-invasive stimulation studies (Miall et al., 2007; Lesage et al., 2012), and psychophysics studies (Lang and Bastian, 1999; Nowak et al., 2004, 2007) in human.

An internal forward model plays a role not only in online control but also in motor learning (Sokolov et al., 2017). In addition to delay compensation, the computation of state prediction has been proposed to serve motor control for cancellation of sensory effects of movements (Blakemore et al., 1998), the transformation between sensory errors and motor errors (Jordan and Rumelhart, 1992), and mental practice for a selection among possible actions (Gentili et al., 2006). A predicted consequence of a movement is to be compared with the actual consequence of that movement, referred to as a prediction error. Note that the prediction error differs from a target error, which is a difference between the target and the actual reaching point. A clever experiment revealed that the prediction error, but not the target error, drives motor adaptation in a visuomotor rotation experiment by dissociating a prediction error from a target error (Mazzoni and Krakauer, 2006). In this experiment, subjects were instructed that a movement of the cursor on the display was rotated counter-clockwise from movement directions of the hand and that they should aim an adjacent target (or intended target) located clockwise from a target of the task (or task target) to intentionally cancel the imposed counter-clockwise rotation. In this design, the predicted outcome of movement was the adjacent target. Therefore, the target error was the difference between the task target and actual movement, while the prediction error was the difference between the intended target and actual movement. By strategically aiming at the adjacent target, the target error was null. A surprising finding was that the target error increased in subsequent trials even when the target error was almost null, indicating that the prediction error, not the target error, drove the adaptation to visuomotor rotation. Intriguingly, similar prediction-based learning algorithms have been also proposed in reinforcement learning where a reward drives a learning process (Barto et al., 1983). There, a reward prediction error (i.e., an actual reward minus an expected reward) but not a reward itself plays a critical role in driving learning processes. In sum, the brain predicts a consequence of its action, compares the prediction with an actual consequence, and improves the next action both in error-based motor learning and reward-based reinforcement learning (Shadmehr et al., 2010).

A less well-known but equally important function of an internal forward model is its role in the computation of gains in feedback control and Kalman filtering. In addition to the predictive computation reviewed above, the forward model is required for the computation of gain matrices both for the feedback controller and Kalman filtering. The optimal feedback control (OFC) model provides a unified framework that integrates diverse computational processes in motor control and motor learning such as the internal forward model, Kalman filter, and feedback control (Todorov and Jordan, 2002; Todorov, 2004). In the OFC model, the cost function (typically expected movement error plus control cost) is optimized so that a task goal is achieved with a minimum energy. There, the state of the body is not directly observable but must be estimated from sensory feedback signals, and the feedback controller is driven by the estimated state. Therefore, the OFC model naturally incorporates the predictive computation of the internal forward model.

The core element in the OFC model is the computation of two gain matrices (Kalman gain and feedback gain matrices). First, the Kalman filter is a recursive method to estimate the current state that integrates a predicted state from an internal forward model and sensory afferents. An optimal estimator is a weighted sum of a predicted state and sensory afferents determined by Kalman gain according to their relative accuracies (or variances). The computation of Kalman gain requires the accuracy of a predicted state from an internal forward model for the optimal tradeoff. Second, the optimally estimated state propels the feedback controller to generate appropriate motor commands, which is a product of the estimated state and the feedback gain matrix. Here, the computation of the feedback gain matrix requires information about the body dynamics and the control cost so that the body is guided to achieve the task goal with a minimum amount of energy. Again, the internal forward model contributes to the computation of feedback gain. Accordingly, the computation of gain matrices in the OFC model necessitates the knowledge of dynamics implemented in an internal forward model.

To summarize, an internal forward model plays at least the three key roles in motor control and motor learning: (1) state prediction for compensating the delay in sensory feedback in online motor control; (2) state prediction for computing a prediction error between a predicted outcome and an actual outcome in motor learning; and (3) computation of Kalman gain and feedback gain. Theoretically speaking, it is possible that these roles are solved collectively by a single, unified forward model or separately by multiple, distributed forward models. Convergent lines of evidence suggest that the cerebellum is a neural correlate of an internal forward model, as discussed below.

### Previous Evidence for Cerebellar Forward Model in the Cerebellum

An internal forward model contributes not only to state prediction for a fast movement but also to the computation of prediction error for motor learning and of gain matrices for the OFC model. A wide range of previous studies converge to the cerebellum processes as the loci of internal forward models (Nowak et al., 2004; Bastian, 2006; Morton and Bastian, 2006; Nowak et al., 2007; Tseng et al., 2007; Lesage et al., 2012). The internal-forward-model hypothesis of the cerebellum indicates that the delayed state and control signal in Figure 1A corresponds to the MFs in Figure 1B and that the predicted state in Figure 1A corresponds to the DNCs in Figure 1B. The cerebellar circuit is characterized by two specific features (Figure 1B): (1) the mostly feedforward connectivity from MFs as an input to DN as an output; and (2) the expansion from MFs (input to the cerebellum) to granule cells (GCs; input to the cerebellar cortex) and the compression from MFs to DNCs (output from the cerebellum). A possible computational role of the expansion coding in GCs is reviewed in Sanger et al. (2020). Given the multitude of computational functions of the internal forward model, it is not surprising that an impairment of the cerebellum leads to a plethora of motor deficiencies collectively known as cerebellar ataxia (Holmes, 1917). Also, cerebellar patients suffer from an inability in motor adaptation and motor learning (Martin et al., 1996; Smith and Shadmehr, 2005; Morton and Bastian, 2006; Tseng et al., 2007). Therefore, the proper functioning of the cerebellum is required for well-coordinated movements and adaptive motor learning.

Most supporting evidence for the internal-forward-model hypothesis of the cerebellum comes from clinical studies, human neuroimaging and non-invasive stimulation studies (Imamizu et al., 2000; Miall et al., 2007; Ishikawa et al., 2016). These studies are broadly categorized into two aspects of the hypothesis: predictive activities and motor learning. First, predictive activities are diminished or altered when the cerebellum is impaired or suppressed. Nowak et al. (2004, 2007), for example, reported that patients of cerebellar agenesis did not show predictive muscle activities in one hand when catching a ball released by the other hand. Miall et al. (2007) applied transcranial magnetic stimulation to the cerebellum during hand movements and found that the hand trajectories deviated from a target. This deviation of trajectories was interpreted as a temporary disruption of forward-model prediction by the stimulation. Second, motor leaning is deteriorated in cerebellar patients. Martin et al. (1996) reported that motor adaptation to displacement prism was severely diminished in cerebellar patients. More recently, Tseng et al. (2007) reported that cerebellar patients had selective impairment in a rapid adaptive process but retained a slow adaptive process. These deficits in motor prediction and motor leaning are supportive of the internal-forward-model hypothesis of the cerebellum.

## Generation of Outputs from The Cerebro-Cerebellum

The DN is the final output station from the cerebro-cerebellum. To contribute to the three roles of an internal forward model mentioned above, DNCs should be able to generate dynamic patterns of output. Our previous study unveiled a simple mechanism to explain a wide range of modulation (i.e., facilitation and suppression) of DNC activity (Ishikawa et al., 2014) and solved the controversy over the generation of burst activity of DNCs before limb movement (Thach, 1970; Strick, 1983; Wetts et al., 1985; Chapman et al., 1986; Fortier et al., 1989; van Kan et al., 1993; Goodkin and Thach, 2003) without a major excitatory drive.

To explain the excitation of DNCs [specifically, deep cerebellar nuclei (dCN) cells], two physiological mechanisms have been proposed. One mechanism is the recruitment of a post-inhibitory rebound excitation (Aizenman and Linden, 1999; Hoebeek et al., 2010; Tadayonnejad et al., 2010; Witter et al., 2013). Another mechanism is the suppression of PCs that facilitates dCN cells by a release from tonic inhibition from PCs, a mechanism known as disinhibition (Albus, 1971; Miyashita and Nagao, 1984; Nagao, 1992; Shinoda et al., 1992; Medina and Mauk, 2000). To address how DNCs become excited or inhibited during voluntary limb movements, we compared the temporal patterns of activity for PCs and DNCs recorded from the same monkeys during step-tracking movements of the wrist (Ishikawa et al., 2014). If the post-inhibitory rebound excitation serves for the facilitation, phasic excitation of PCs and a concomitant inhibition of DNCs should precede the main excitation of DNCs. On the other hand, if the disinhibition serves for the facilitation, we should observe the suppression of PCs and the activation of DNCs at the same timing. We found that (Ishikawa et al., 2014) the majority of PCs in the Cerebro-cerebellum demonstrated suppression before the onset of wrist movements. At the same time, the majority of DNCs were activated without prior suppression. This finding supports the disinhibition mechanism that the movement-related activation of DNCs occurs when they are released from tonic inhibition from PCs.

**Figure 2:**
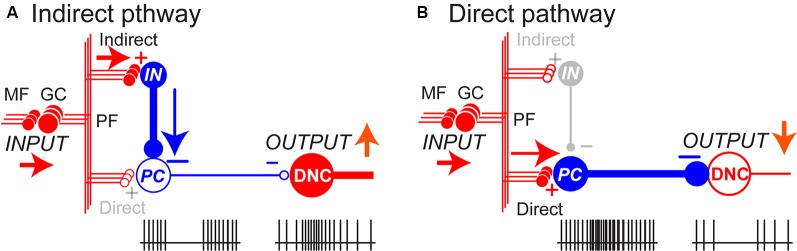
The balance between parallel pathways in the cerebellar cortex determine DNC output. **(A)** In the indirect pathway, the PC activity is suppressed through inhibitory interneurons (IN), which in turn enhances the DNC activity with disinhibition. **(B)** In the direct pathway, PC is activated through excitatory parallel fiber (PF) inputs, which in turn suppresses the DNC activity. The balance between the two pathways determines the final output patterns of individual DNCs. In this way, inhibitory PCs can exert bidirectional effects on DNCs and generate a variety of output patterns. Pluses (+) and minuses (−) represent excitatory and inhibitory synapses, respectively. Adapted from Ishikawa et al. (2014) under CC-BY license.

The proposed mechanism in generating burst activities of DNCs is summarized in Figure 2. MF inputs to the cerebellar cortex are relayed by GCs and activate one or both of parallel pathways to PCs. The indirect pathway suppresses PCs *via* inhibitory interneurons (INs; Figure 2A) whereas the direct pathway activates PCs directly through parallel fibers (PFs; Figure 2B). In this way, the activity of individual DNC is regulated by the summation of inputs through the parallel pathways. In line with our proposal, Dean and Porrill (2010) proposed that the parallel pathways perform in a competitive way to suppress or facilitate the activity of PCs in the cerebellar cortex. Our finding extends their idea to explain the role of the parallel pathways for the generation of dynamic output from DN (Ishikawa et al., 2014). Overall, in our population of PCs, movement-related suppression of simple spike (SS) activity dominates before movement onset and contributes to the initiation of movement, while movement-related facilitation dominates after movement onset and contributes to termination of movement (see Figures 8A, 9A in Ishikawa et al., 2014).

The differential recruitment of the two pathways is also organized in a spatially congruent manner; activities of DNCs were modulated by activities of PCs with overlapping receptive fields (RFs; Ishikawa et al., 2014). A large proportion of PCs whose somatosensory RFs were found in the distal arm (i.e., around the wrist joint) showed strong suppression before movement onset, whereas the majority of DNCs with the same RFs showed a concurrent burst of activity. In contrast, PCs with RFs in the proximal arm demonstrated a marked and simultaneous increase in activity, while DNCs with the same RFs were strongly suppressed. Our observation suggests that activation of DNCs by disinhibition from PCs facilitates the execution of wrist movement, whereas suppression of the DNCs due to increased PC activity contributes to the stabilization of proximal muscles and improves task performance.

The organization of the parallel pathways of the cerebellar outputs and its spatially congruent recruitment reminds us of two often overlooked clinical signs described by Holmes (i.e., *asthenia* and *adventitiousness*). We proposed that these signs may be caused by malfunctions of the two output modes (Ishikawa et al., 2015). Asthenia represents a failure of the recruitment of muscle activities resulting in delayed initiation and the slow build-up of movement, whereas adventitious movement is sporadic or erratic activation of muscles to be suppressed resulting in instability. Namely, the asthenia and adventitiousness may reflect deficits in the control of the disinhibition and inhibition, and they could be essential building blocks of various ataxic movements (Ishikawa et al., 2015). Overall, the malfunction of the parallel pathways disturbs whatever contribution of the cerebellum by altering the proper input-output organization.

## Functional Evidence for Cerebellar Forward Model

### Neural Evidence for Cerebellar Internal Model

In contrast to the wealth of evidence, it is intriguing that few previous studies hitherto examined the internal-forward-model hypothesis of the cerebellum from electrophysiological data. Of particularly notable is a series of electrophysiological studies recorded from monkeys performing a manual pursuit tracking task (Roitman et al., 2005; Pasalar et al., 2006; Ebner and Pasalar, 2008). Firing rates of SSs of PCs reflected and preceded movement kinematics (hand position and velocity) irrespective of assistive/resistive forces imposed on the hand, supporting that the PCs implement the forward-model computation in the kinematic space. On the other hand, there is another electrophysiological study that contradicts with the internal-forward-model hypothesis. Firing rates of PCs recorded from monkeys performing an elbow flexion/extension task consistently covaried with the level of imposed force, indicating that the PCs represent movement dynamics but not movement kinematics, the findings more consistent with the internal-inverse-model computation (Yamamoto et al., 2007). Therefore, it is yet an open question whether the cerebellum performs the forward-model computation in kinematic space or the inverse-model computation in dynamic space. These studies posit that kinematic and dynamic representations in PCs correspond to a forward model or an inverse model, respectively. But both internal models contain kinematic and dynamic representations, and PCs are not final outputs of the cerebellum. We hence think that examining the coding representation of PCs does not directly address the question about the internal-model hypotheses of the entire cerebellum.

Instead of focusing on a neural representation in single population as in the previous studies, our recent study tackled this problem by examining how neural representations are transformed from one population to another through the cerebellar circuit (Tanaka et al., 2019). The cerebellum has the unique anatomical structure composed of feedforward connectivity particularly suited for the internal-forward-model computation (Figure 1B). One natural prediction derived from the hypothesis is that a current output of an internal forward model should contain predictive information about a future input to that internal forward model. If the internal-forward-model hypothesis of the cerebellum holds, current activities of the cerebellar outputs should be able to predict future activities of the cerebellar inputs. Our previous studies reported movement-related modulation of firing rates of MFs, Golgi cells, PCs and DNCs recorded from monkeys performing step-track wrist movements (Ishikawa et al., 2014; Tomatsu et al., 2016). Specifically, we analyzed the firing rates of 94 MFs, 83 PCs, and 73 DNCs (Tanaka et al., 2019).

We first addressed how the firing cells of PCs were driven by the firing rates of MFs. A linear weighted sum of MF firing rates reconstructed PC firing rates most parsimoniously, in comparison with a thresholding model, a quadratic model or an FIR model. The successful reconstruction by the linear model was unanticipated given the fact that a PC receives an estimated 10^5^ PF inputs whereas our linear reconstruction included only 94 MF inputs per PC. This was probably because all recorded cells were task-related whose activities were modulated by the movement task, and we surmise that only a few dozens of MFs contributed significantly to the task-related firing rates of PCs. Similarly, we found that the firing rates of DNCs were also well reconstructed as a weighted linear sum of MFs and PCs. Dominant computational models of the cerebellar cortex posit more complex processes; the perceptron model assumes nonlinear thresholding at PCs (Marr, 1969; Albus, 1971), and the adaptive filter model assumes a dependence of PC firing rates on current and previous MF firing rates (Fujita, 1982; Dean et al., 2010). Our analysis, on the other hand, implies that the cerebellar computation is rather linear, markedly simpler than previously thought.

We then proceeded to directly test the internal-forward-model hypothesis by examining whether the cerebellar output could predict the cerebellar input in the future. Following the linear reconstructions of PC and DNC firing rates, the MF firing rates at time *t*+*t*_1_ were predicted as a linear weighted sum of the DNC firing rates at time *t*. We found that the linear prediction of MF firing rates was statistically significant when compared to directionally randomized surrogate data. This analysis suggests that the current output from the cerebellum (DNC firing rates at time *t*) contained predictive information about the future input to the cerebellum (MF firing rates at time *t*+*t*_1_), which in turn supports the internal-forward-model hypothesis of the cerebellum. Since the MF activities analyzed here originate from the motor cortex, the cerebellar output predicts the future state of the motor cortex, which in turn returns to the motor cortex through the thalamus.

**Figure 3:**
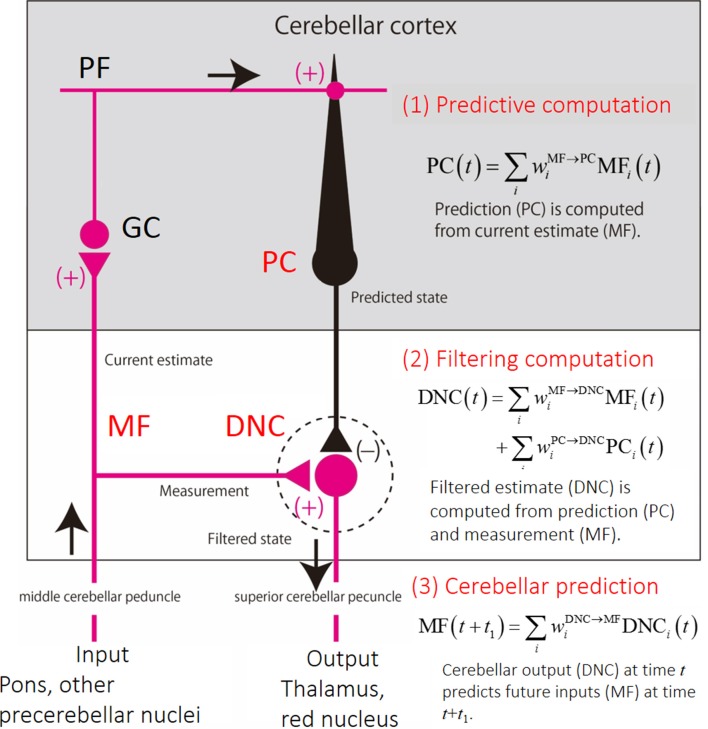
Schematics of the cerebellar circuit and corresponding computational steps. (1) Prediction computation. The PCs computes a predicted state from a current estimate conveyed by the MFs. (2) Filtering computation. The predictive state computed by the PCs are integrated with an observation signal conveyed by other MFs for optimal estimation in DNCs. (3) Cerebellar prediction. The current output from the cerebellar circuit (DNCs) can predict future inputs to the cerebellum (MFs).

We note that the linear equations derived from the experimental firing rates resemble those of optimal estimation known as Kalman filter. Based on formal correspondence between the experimentally derived linear equations and the Kalman-filter equations, we speculate the following computational steps in the cerebellar circuits (Figure 3): (1) the PCs compute a predictive state from a current estimate conveyed by the MFs (Predictive computation); (2) the DNC activities combine the predicted state from the PC activities and sensory feedback from the MF activities (Filtering computation); and (3) the DNC activities predicts a future input to the MFs (Cerebellar prediction). Our finding indicates that the cerebellum performs not only an internal-forward-model prediction but also an optimal integration of a predicted state and sensory feedback signals.

### Behavioral Evidence for Cerebellar Internal Model

**Figure 4:**
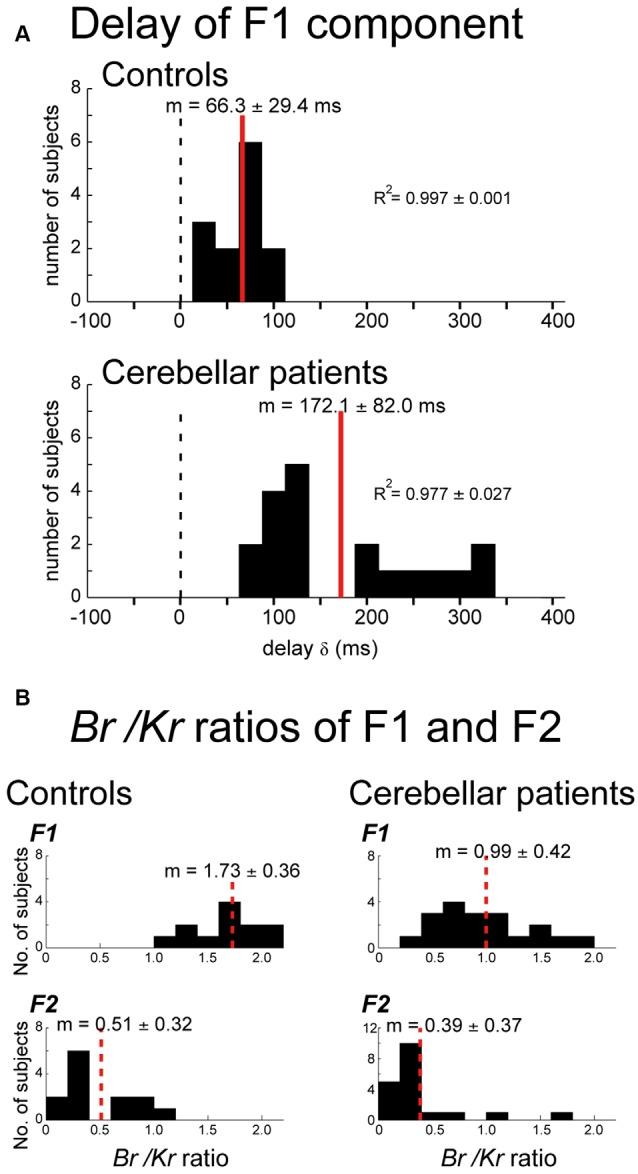
Changes in movement kinematics of ataxic patients. **(A)** Delay of the F1 domain of the wrist movement from the target motion. Cross-correlation was calculated by changing the delay δ of the target motion relative to the wrist movement. *Controls*: Histogram of the optimal delay δ for the control subjects (*n* = 13). *Cerebellar patients*: Histogram of the optimal delay δ for the cerebellar patients (*n* = 19). **(B)** Comparison of the *B*_r_/*K*_r_ ratios for the F1 and F2 components between the controls and the cerebellar patients. *Controls*: *B*_r_/*K*_r_ ratios of the control subjects for the F1 component (top) and the F2 component (bottom; *n* = 13). Note the highly significant difference between the two components. *Cerebellar patients*: *B*_r_/*K*_r_ ratios of the patients for the F1 (top) and the F2 (bottom) components (*n* = 19). Note the selective decrease of *B*_r_/*K*_r_ ratios for the F1 component in the patients. Adapted from Kakei et al. (2019) under CC-BY license.

Although the cerebro-cerebellum has long been suggested as a neural substrate of internal forward models (Wolpert and Miall, 1996; Bastian, 2006; Ebner and Pasalar, 2008; Ishikawa et al., 2016), few behavioral methods are available to evaluate the contribution of forward models in patients with cerebellar ataxia (but see Bhanpuri et al., 2014). Our previous studies have developed a method to analyze the contribution of position- and velocity-dependent motor commands (i.e., muscle activities) in visually-guided pursuit movements of the wrist (termed as *B*_r_/*K*_r_ ratio, defined below; Lee et al., 2015; Kakei et al., 2019). Here, *B_r_* and *K_r_* stand for viscous and elastic coefficients, respectively, estimated from wrist movements of a subject with a canonical correlation analysis, and the *B*_r_/*K*_r_ ratio evaluates the ratio of velocity control to position control. The movement kinematics of wrist was decomposed into a slower (i.e., lower-frequency, <0.5 Hz) F1 component and a faster (i.e., higher-frequency, >0.5 Hz) F2 component, and the *B*_r_/*K*_r_ ratio was computed for F1 component and F2 component, respectively. The F1 component belonged to the same frequency range of the target motion and encoded both velocity and position (higher *B*_r_/*K*_r_ ratio) of the target motion. This kinematic formula of the F1 component appeared optimal to synchronize the wrist movement with the target motion in a *predictive* manner. Indeed, for the control subjects, the F1 component lagged behind the target motion for about 60 ms (15.0–107.4 ms, mean ± SD = 66.3 ± 29.4 ms, 13 subjects; Figure 4A, Controls). The short delay excludes a possibility that the F1 component of the wrist movement was generated with visual feedback of the target motion. The conduction time of the peripheral motor nerve (~10 ms) and electromechanical delay (~50 ms) alone would take that long (~60 ms). Thus, the delay of the F1 component was too short to be a feedback delay. Rather, the generation of the F1 component in the CNS must have preceded the corresponding motion of the target, considering the average lead time of neuron activity in the primary motor cortex for the wrist movement (~100 ms).

We next evaluated the delay of the F1 component in patients with cerebellar ataxia. The F1 component was delayed significantly more (~100 ms) in the patient group (79.5–322.4 ms, mean ± SD = 172.1 ± 82.0 ms, 19 subjects; *p* < 0.0001) than in the control group (Figure 4A, Patients). The delay may be explained as poor recruitment of facilitation in DN due to a decrease in the disinhibition of DNCs, i.e., asthenia (Ishikawa et al., 2015). The prediction delayed by this amount is no longer predictive and may force the patients to rely on the pure feedback control, further destabilizing the wrist movement ataxic (Kakei et al., 2019).

We further demonstrated that the *B*_r_/*K*_r_ ratios of the predictive (F1) component had a significant difference between the control and patient groups. Namely, the *B*_r_/*K*_r_ ratios of the F1 component of the patient group (0.3–1.9, mean ± SD = 0.99 ± 0.42; Figure 4B, Patients, F1) were significantly lower than those of the control group (1.4–2.5, mean ± SD = 1.73 ± 0.36; Figure 4B, Controls, F1; *p* < 0.001), suggesting difficulty in recruiting velocity control in cerebellar patients. In contrast, *B*_r_/*K*_r_ ratios of the F2 component were comparable for both groups (compare Figure 4B, *Cerebellar patients, F2* and *Controls, F2*). Taken together, our results support the hypothesis that cerebellar patients have an impairment in the forward-model prediction while relatively maintaining corrective control in response to sensory feedback.

**Figure 5:**
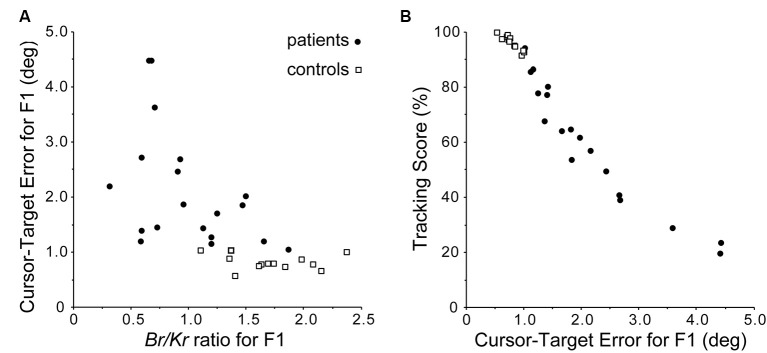
Accuracy of predictive (F1) component. **(A)** Relationship between the *B*_r_/*K*_r_ ratios for F1 component and Cursor-Target error for F1 (F1 error, in short). The F1 error is defined as an average error between the target motion and the F1 component of the movement during a trial. Note the negative correlation. **(B)** Relationship between F1 error and Tracking Score. Note the linear relationship. Overall, *B*_r_/*K*_r_ ratio for the F1 component has a strong positive correlation with the accuracy of the pursuit movement. Adapted from Kakei et al. (2019) under CC-BY license.

We then proceeded to examine the relationship between *B*_r_/*K*_r_ ratios of the F1 component and performance/accuracy of pursuit movement in the cerebellar patients and the control subjects (Figure 5), because the characteristic decrease in *B*_r_/*K*_r_ ratio of the F1 component might have reflected impaired predictive control in the patient group. To test this hypothesis, we examined the relationship between the *B*_r_/*K*_r_ ratios of the F1 component and the accuracy of the pursuit movement (i.e., F1 error, Kakei et al., 2019). The *B*_r_/*K*_r_ ratio of the F1 component and the F1 error were negatively correlated (Figure 5A). Next, we examined the relationship between the F1 error and the tracking score. The tracking score was defined as a percentage of time when the cursor was kept within the target circle. The F1 error and the tracking score demonstrated a strikingly linear, negative correlation (Figure 5B). Overall, the *B*_r_/*K*_r_ ratio of the F1 component is a useful measure to assess the accuracy of predictive control, which could be quantified noninvasively in a clinical setting.

## Anatomical Structure Supporting for Cerebellar Forward Models

### Morphologic Substrata of the cerebro-Cerebellum for Kalman Filter

**Figure 6:**
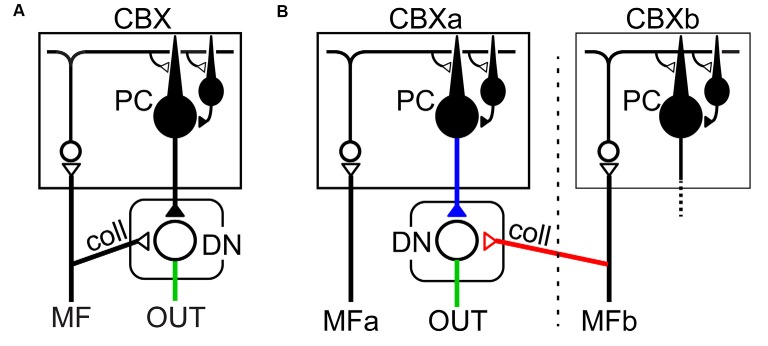
Two schematics of cortico-nuclear organization. **(A)** The conventional scheme in which the same MF projects to the cerebellar cortex (CBX) and DN both of which belong to the same corticonuclear complex (Ito, 1984). **(B)** Proposed scheme that one MF (MFa) from pontine nuclei (PN) projects to the cerebellar cortex (i.e., cerebro-cerebellum; CBXa) without collateral projection to DN, whereas another, separate MF (MFb) projects to DN. Note that MFa and MFb have distinct projection areas in the cerebellar cortex, CBXa, and CBXb, respectively. This scheme is consistent with the requirements of the Kalman-filter model.

Here we argue morphological substrata supporting our findings of predictive computation presented in Section “Behavioral Evidence for Cerebellar Internal Model”. The conventional circuit diagram of the cerebro-cerebellum (Figure 6A) depicts the MFs projecting both to the cerebellar cortex and DN as collaterals, implying that the cerebellar cortex and DN share the same source of input. On the other hand, the prerequisite of the Kalman filter is the two distinct inputs: current estimate and current measurement; One MF input comes from the cerebral cortex to the cerebellar cortex (*via* PN) and plays an essential role in the prediction step, and another MF input to DN conveys sensory feedback information and plays a critical role in the filtering step. Therefore, the conventional diagram of the cerebro-cerebellum in which the same MF projecting to the cerebellar cortex and DN is not compatible with our proposal of Kalman-filter computation in the cerebellum.

Discordant to the conventional diagram, extant anatomical studies rather suggest that the cerebro-cerebellum receives respective projections to the cerebellar cortex and DN (Figure 6B). The first requirement of the Kalman-filter model is that MFs originated from the PN project to the cerebro-cerebellum *without* collaterals to DN. Indeed, Kelly and Strick (2003) demonstrated a strong projection from the primary motor cortex (M1) to the cerebro-cerebellum, which was assumed to carry efference copy signals. Also, Na et al. (2019) demonstrated that MFs originated from PN have virtually no collateral projection to DN on their way to the cerebro-cerebellum. Taken together, it is most likely that the first requirement is satisfied for the input from M1 to the cerebro-cerebellum. The second requirement of the Kalman-filter model is that MFs conveying feedback input provide collaterals to DN. Wu et al. (1999) demonstrated that MFs originated from the lateral reticular nucleus (LRN), which receives strong somatosensory inputs from the spinal cord, have abundant collateral projection to DN and other cerebellar nuclei on their way to the vermis and the intermediate zone (see Figures 8–10 in Wu et al., 1999). Also, these MFs from PN and LRN have only minor overlap in their projection to the cerebellar cortex (Wu et al., 1999; Na et al., 2019). These observations converge to the neural organization compatible with the requirement of the Kalman-filter model (Figure 6B).

Consistent with the anatomical observations, there were two functionally distinct populations of MFs in our data set (Tanaka et al., 2019). One population of MFs contributed to the prediction step and the other population of MFs contributed to the filtering step. We confirmed that partially distinct populations of MFs contributed to the reconstructions of PCs and DNCs, respectively; the average correlation coefficient between weights of MF–PC and MF–DNC projections was no more than 0.060. A statistical test based on resampling verified that the correlation between the two MF populations was statistically significant (*p* < 10^−5^). Therefore, it was concluded that PCs and DNCs received the projections from distinct populations of MFs, thereby fulfilling the requirements of the Kalman-filter model.

As outlined in the “Introduction” section, an input to and an output from a specific region of the cerebellum are a key to understanding of the function played by that region. The “corticonuclear organization” depicted in Figure 6B appears to be specific for the cerebro-cerebellum (i.e., the *newer* part of the cerebellum), the other regions of the cerebellum could receive input projections in different ways (Ito, 1984). For instance, in the vestibular nucleus, nuclear neurons may act as a relay for MF afferents, whereas PCs activated by the MF afferents may exert modulatory action on the nuclear relay cells (Figure 6A, see also Figure 92A in Ito, 1984). Similarly, in the fastigial nucleus, nuclear cells may serve as a relay for PC output reflecting MF inputs, while collaterals of MFs provide a background excitation on which PCs can impose efficient bidirectional modulation (i.e., inhibition and disinhibition; Figure 6A, see also Figure 92B in Ito, 1984). In these cerebellar regions, the corticonuclear organization is not compatible with the Kalman filter, where the cerebellar cortex and DN receive shared MF projections. Overall, even if the input-output organization is common for the entire cerebellar cortex, different regions may contribute to computationally different operations depending on the organization of MF collaterals in the cerebellar nuclei (Ito, 1984). It should be noted that Ito (1984) pointed out the possibility of this type of heterotopic combination of a direct collateral MF input and an indirect MF input *via* PCs (i.e., “sidepath”) to DN (see Figure 92D in Ito, 1984), to explain spontaneous activities of DN neurons that lack collateral inputs of MFs originated from PN (Allen and Tsukahara, 1974).

### Compressed Prediction of the Cerebellar Internal Model

The cerebro-cerebellar loop has more abundant projections from the cerebral cortex to the cerebellum than those from the cerebellum to the cerebral cortex (Figure 1B). To the best of our knowledge, little attention is paid to the asymmetry of the cerebro-cerebellar loop, in terms of the number of output neurons on each side of the loop. The number of axons in the cerebral peduncle (CP) conveying cortical outputs to PN and other precerebellar nuclei is estimated as twenty-one million in humans (Tomasch, 1969). On the other hand, the number of axons in the return path, i.e., the superior cerebellar peduncle (SCP) relaying the cerebellar output to the thalamus, is no more than 0.8 million in human (Heidary and Tomasch, 1969). Therefore, the output from DN can convey less than 5% of the information of the cortical output assuming comparable discharge frequencies for corticofugal neurons and DNCs (Kakei et al., 1999, 2001; Ishikawa et al., 2014; Tomatsu et al., 2016). The cerebro-cerebellum returns its output back to the cerebral cortex that is considerably compressed from the input it receives. One may then wonder what the functional advantage of the compact representation is. There appears no consensus on the functional role of the compact representation (Sanger et al., 2020). Given the fact that the cerebellum contributes to fast, trained and automated motor control with reduced effort and attention, the compressed representation may be beneficial or even necessary to extract relevant information from numerous and redundant cerebellar inputs and to assign more attention to the task currently in the focus.

## Cognitive Functions and Cerebellar Forward Models

The cerebellum was once thought of as an organ for motor coordination. The motor cerebellum is mainly represented in the anterior lobe with a smaller, secondary representation in lobule VIII (Kelly and Strick, 2003). Although possible involvement of the cerebellum in non-motor mental functions had been suggested occasionally in the past, it has become the subject of systematic consideration since the beginning of 1990s (Leiner et al., 1986; Schmahmann, 1991; Ito, 1993; Schmahmann, 2004; Ito, 2008). With a use of trans-neuronal transport of neurotropic viruses, Middleton and Strick (1994) provided the first evidence in the monkey that the cerebellum is connected to the non-motor area 46 of the prefrontal cortex and revised the conventional view of the cerebellar devotion to motor control. Strick and his colleagues eventually established that the hemispheric parts of the cerebellum (mainly Crus I and Crus II) are connected with various cortical association areas through DN (Middleton and Strick, 1994; Dum and Strick, 2003; Kelly and Strick, 2003).

The existence of the human non-motor cerebellum was later demonstrated repeatedly in non-invasive imaging studies (e.g., Hanakawa et al., 2003; for reviews see Stoodley and Schmahmann, 2009; Buckner, 2013). A surprisingly powerful approach capable to map the topographical organization of the cerebellar cortex in the human has recently provided insight into the functional mapping between the cerebellum and the cerebral cortex. The approach derives from the observation that brain organization can be inferred by measuring spontaneous low-frequency fluctuations in intrinsic activity (Biswal et al., 1995; Fox and Raichle, 2007). Recently, Buckner et al. (2011) and Wang et al. (2013) demonstrated base on this approach that the cerebro-cerebellum collects information from almost all the entire cortical areas, suggesting that the cerebellum still keeps the position of the CNS *hub* even in human. More recently, Guell et al. (2018) used data from the Human Connectome Project (*n* = 787) to analyze cerebellar fMRI task activation (motor, working memory, language, social and emotion processing) and resting-state functional connectivity. They demonstrated novel aspects of the functional topography of the human cerebellum. There were two distinct representations (lobules I-VI and lobule VIII) of motor activation that was consistent with prior studies, in particular with the above-mentioned trans-neuronal tracing study in the monkey (Kelly and Strick, 2003). Newly revealed were three distinct regions (Crus I, Crus II, lobules IX/X) in the cerebellar posterior lobe that show topographical relationship with cortical association areas (Buckner et al., 2011; Wang et al., 2013). Each region contains four domains of non-motor functions (working memory, language, social, and emotional task processing). Indeed, lesions in the posterior lobe result in the cerebellar cognitive affective syndrome (CCAS), which includes deficits in executive function, visual spatial processing, linguistic skills and regulation of affect (Schmahmann, 2019).

The critical question arises whether the Kalman-filter mechanism identified for the prediction in the *motor* part of the cerebro-cerebellum (Tanaka et al., 2019) generalizes to the *cognitive/affective* part of the cerebro-cerebellum. Our dataset of MFs, PCs, and DNCs recorded during the motor task cannot support or reject the predictive mechanism of the cerebellum for cognitive/affective functions. Nevertheless, it is possible to search for the *same* corticonuclear organization (Figure 6B) in the *non-motor* part of the cerebro-cerebellum. There are two requirements: (1) the main MFa input to the cerebro-cerebellum is originated from a non-motor area and relayed by PN, and (2) the filtering MFb input is originated from a distinct cortical or subcortical source and relayed by a *non-PN* nucleus that has significant collateral projection to DN (Figure 6B). The requirement 2 is the key because the requirement one is common for several cortical areas, including prefrontal areas (Schmahmann and Pandya, 1997), parietal association areas (Schmahmann and Pandya, 1989), superior temporal areas (Schmahmann and Pandya, 1991), occipitotemporal and parahippocampal areas (Schmahmann and Pandya, 1993). There are only a few major sources of collateral MF inputs to DN, the lateral reticular nucleus (LRN; Wu et al., 1999) and the nucleus reticularis tegmenti pontis (NRTP; Gerrits and Voogd, 1986) in the reticular formation. The LRN receives main inputs from the spinal cord (Alstermark and Ekerot, 2013) and additional inputs from the sensorimotor areas and the red nucleus (Bruckmoser et al., 1969; Matsuyama and Drew, 1997). The NRTP receives inputs mainly from the sensorimotor areas, the prefrontal areas and the parietal association areas (Schmahmann et al., 2004). Overall, the Kalman-filter model is at least compatible with the non-motor part of the cerebro-cerebellum if the two inputs to non-motor parts of DN (Dum and Strick, 2003) are proven to have different origins in future studies.

## Remaining Issues About Cerebellar Internal Models

This review article has discussed physiological, behavioral, and morphological evidence supporting the internal-forward-model hypothesis of the cerebellum, with an emphasis on our recent studies. This final section expands our speculation on a possible computational role of the cerebro-cerebellar loops and raises remaining unsolved issues on the functioning of the cerebellum for a future study.

### Possible Computational Role of Cerebro-Cerebellar Loops

The cerebral cortex and the cerebellum have evolved together as their volume has increased in a proportional manner (Rilling and Insel, 1998), and form an anatomically closed connectivity known as the cerebro-cerebellar loop (Kelly and Strick, 2003; Bostan et al., 2013). These findings indicate that the evolutionally conserved anatomical structure composed of the cerebral cortex and the cerebellum plays a functionally relevant role, but to the best of our knowledge, there appears no consensus about its specific function. We below expand our speculation on a computational role considering our findings and artificial neural networks.

The cerebral cortex and the cerebellum have contrasting anatomical structures. The cerebral cortex is characterized by hierarchical recurrent connections composed of local connections across cortical layers and lateral connections (for a review see DeFelipe and Jones, 1988), so it is appropriate to model the cerebral cortex, at least locally, as a recurrent neural network. An artificial neural network model with recurrent connections is known to be able to approximate any dynamical systems (Funahashi and Nakamura, 1993), so it is appropriate to model a neural process that evolves over a period such as a production of a motor sequence. A recurrent neural network is also known to be difficult to train and control because it may exhibit chaotic behavior (Sompolinsky et al., 1988; Sussillo and Abbott, 2009; Laje and Buonomano, 2013; Ben-Shushan and Tsodyks, 2017). On the other hand, the cerebellum consists essentially of a feedforward connectivity from the MFs as an input to the cerebellar nuclei as an output. An artificial neural network model composed of more than two layers is known to be able to approximate any continuous mapping (Cybenko, 1989; Funahashi, 1989; Hornik et al., 1989), which is a theoretical basis of the universal cerebellar transform (Schmahmann, 2004). Also, the MF inputs are considerably expanded by the GCs by about one-thousand-fold and then converged into the DNCs (Figure 1B). This divergence-convergence structure resembles a shallow learning scheme in machine learning. A feedforward neural network model is straightforward to train, but its computation is limited to a static input-output function.

**Figure 7:**
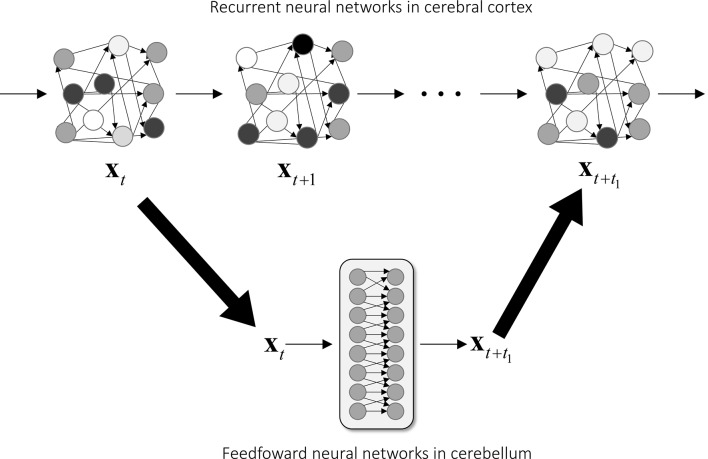
Proposed mechanism of predictive stabilization of cortical dynamics through the cerebro-cerebellar loop. The dynamics of the cerebral cortex and the cerebellum are modeled as a recurrent neural network (top row) and a feedforward neural network (bottom row), respectively. The cerebellum receives a current cortical state at time *t* and projects back a future cortical state at time *t*+*t*_1_ through the cerebro-cerebellar loop. In this proposed mechanism, the cerebellar prediction guides and stabilizes the recurrent dynamics of the cerebral cortex, thereby realizing fast and robust information processing.

We here speculate a computational possibility that the cerebellum copies the dynamics of the cerebral cortex and then predicts the state of the cerebral cortex for fast and stable operations in motor control and cognitive processing (Figure 7). There are some advantages and disadvantages of recurrent and feedforward neural networks, and we propose that the recurrent neural networks of the cerebral cortex and the feedforward neural networks of the cerebellum complement each other. Recurrent connections in a neural network provide computational flexibility to model a dynamical system (Funahashi and Nakamura, 1993) but cause a chaotic instability due to dependence on previous activities and random noises (Sompolinsky et al., 1988). Therefore, a sequence production solely by a recurrent neural network can be unstable against small fluctuations in activities and unwanted noises (Jaeger and Haas, 2004). A feedforward neural network, on the other hand, is stable because its output depends not on previous inputs but only current inputs and a fluctuation at one point of time does not propagate over time. Our recent results demonstrated that the cerebellar circuit could perform the predictive computation of an internal forward model, so we propose that the cerebellum tames computationally flexible but chaotic dynamics of cortical recurrent neural network by predicting the expected activity of the cerebral cortex. In line with our proposal, the FORCE learning algorithm generates stable patterns of activity in a recurrent neural network by adding feedback connections from the output unit to recurrent units (Sussillo and Abbott, 2009). In our proposed scheme, the feedforward network of the cerebellum continuously monitors and predicts the activities of the recurrent network of the cerebral cortex. The recurrent network, in turn, stabilizes its activities and corrects any deviations by comparing the current activity in the recurrent network and the predicted activity from the cerebellar feedforward network. Therefore, an internal model allows fast and robust computation not only in motor control but also in recurrent neural networks in the cerebral cortex.

Whereas recurrent and feedforward neural networks differ from each other in their formulation, they are in fact equivalent because recurrent neural networks can be transformed into feedforward neural networks by a proper redefinition. A standard algorithm for training a recurrent neural network, backpropagation-through-time, exploits the fact that a recurrent neural network can be regarded as a temporally unfolded feedforward neural network (Werbos, 1990). Also, it was shown that a proper redefinition of units can transform a recurrent neural network into a feedforward neural network, known as a method of matrix decomposition (Schur decomposition) in matrix algebra (Goldman, 2009). Therefore, recurrent and feedforward neural networks possess the same ability in terms of computation.

Our computational scheme requires that a part of the cerebro-cerebellum and a part of the cerebral cortex connected by a cerebro-cerebellar loop should perform the same computation so that the cerebellum can predict activity patterns of the cerebral cortex. We, therefore, speculate that the computational role of the cerebellum is to copy the function of the cerebral cortex for predicting and stabilizing the dynamics of the cerebral cortex. Results from a recent imaging study are in line with this speculation; activities of layer 5 pyramidal cells in the neocortex and those of GCs in the cerebellar cortex share task-encoding characteristics acquired during learning, indicating that a key function of cerebro-cerebellar loop is the propagation of shared neural dynamics (Wagner et al., 2019). A tentative computational scheme posits that dynamics learned by a recurrent neural network are transferred to a feedforward neural network, and then the feedforward neural network stabilizes the dynamics of the recurrent neural network by predicting the dynamics. An additional merit of feedforward neural network is single-shot, fast computation; a recurrent neural network requires multiple, iterative steps for computing a transition from **x**_*t*_ to **x**_(*t*+*t*_1_)_ (Figure 7, top), which could be computed in a single step in a feedforward network (Figure 7, bottom). Our speculation discussed here is mainly motivated by the results of artificial neural networks; however, given the recent productive interactions between deep neural networks and the primate visual system, it is not unreasonable to incorporate ideas developed in artificial neural networks to understanding the biological nervous system. We hope that this speculative function proposed here will guide the future computational study on the role of cerebro-cerebellar loops.

### Future Problems

Our recent study arguably demonstrated that the cerebellar output (activities of DCs) at current time was predictive about the cerebellar input (activities of MFs) at a future time, supporting the hypothesis that the cerebellum performs the computation of an internal forward model (Tanaka et al., 2019). The dataset analyzed in this study was recorded when the monkey was over-trained for the movement task for years and there was no sign of learning in performance. Hence, our study demonstrated one aspect of forward model, namely the predictive activity, but not another aspect of forward model, namely motor learning. Electrophysiological recording of single units usually requires averages to remove trial variance, so it is not ideal for analyzing activity changes in an individual trial before, during, and after learning. This is particularly problematic when we want to address the circuit-level analysis where cells from multiple populations need to be recorded simultaneously. Fortunately, new recording techniques such as calcium imaging are available to track learning-related changes in the activities of multiple neurons (Wagner et al., 2019). We hope that the new approach will reveal the contribution of the internal-forward-model to the motor-learning shortly.

We would like to conclude this review article by enlisting two unanswered questions that we think important for promoting a better understanding of the computational functions and the neural mechanisms of the cerebellum. First, how is a predictive activity computed in the cerebellum utilized in the cerebral cortex? Our previous study reported that MF activities, the input to the cerebellum, shared response properties with the activity of neurons in motor areas, implying that activities of the cortical neurons are copied into the cerebellum as an input (Tomatsu et al., 2016). On the other hand, it remains to be examined how the cerebellar output influences the activities of the cortical neurons. Second, how do linear dynamics in the cerebellar circuit approximate the nonlinear dynamics of the musculoskeletal system? Biological motor control must face with the large degrees of freedom of the body and corresponding nonlinear dynamics, and we know little about how such dynamics is solved in the brain. We hope that future studies will take a challenge in addressing these questions toward the goal of understanding the functions and the neural mechanisms of the cerebellum.

## Author Contributions

SK, HT, TI and JL conceived the research. HT and SK drafted the manuscript. HT, SK, TI and JL finalized the manuscript.

## Conflict of Interest

The authors declare that the research was conducted in the absence of any commercial or financial relationships that could be construed as a potential conflict of interest.
